# A Novel Analysis Method for Simultaneous Determination of 31 Pesticides by High-Performance Liquid Chromatography-Tandem Mass Spectrometry in Ginseng

**DOI:** 10.1155/2022/4208243

**Published:** 2022-02-16

**Authors:** Xingang Hou, Liangyue Liu, Liping Wei, Da Feng, Meng Lv, Xiumei Wang, Xiaolong Yu, Zhongbin Lu, Zhiguang Hou

**Affiliations:** ^1^College of Plant Protection, Jilin Agricultural University, Jilin 130118, China; ^2^Safety Evaluation Center, Shenyang Research Institute of Chemical Industry, Shenyang 110021, China

## Abstract

Ginseng is a perennial herb with a long growth cycle and is known to easily accumulate pesticides during its growth process, seriously threatening people's health. Therefore, to ensure safe consumption, it is necessary to detect and monitor pesticide residues in ginseng. In this study, a novel analysis method was established for simultaneous determination of 31 pesticides in ginseng by high-performance liquid chromatography-mass spectrometry. Ginseng samples were extracted using acetonitrile, cleaned up by primary secondary amine (PSA) solid-phase extraction column eluted with acetonitrile-toluene, and then detected in multiple reaction mode (MRM). The calibration curves of target compounds were linear in the range of 0.005–1.0 mg/L, with correlation coefficients greater than 0.9921. The limits of detection of all the pesticides in ginseng were between 4.4×10^−5^ and 1.6 × 10^−2^ mg/kg. For fresh ginseng, the average recoveries ranged from 72.1 to 111.6%, and the relative standard deviations were 1.3–12.2%. For dry ginseng, the average recoveries were 74.3–108.3%, and the relative standard deviations were 0.9–14.9%. The residual concentrations of some pesticides in real samples were greater than the maximum residue limit (MRL) for European Union (EU). The method established here is rapid and simple with high sensitivity and good reproducibility, which is sensitive in the residue analysis of many pesticides in ginseng.

## 1. Introduction

Ginseng (*Panax ginseng* C.A. Mey.) is a valuable medicinal plant with a history of more than 2,000 years [1, 2]. Clinical tests have proven that ginseng plays a role in stimulating blood vessels, regulating nerves, increasing appetite, sedating the brain, restoring fatigue, promoting metabolism, enhancing liver detoxification functions, regulating the endocrine system, improving bone marrow hematopoiesis, and strengthening the body's immune response [3–5]. With the enhancement in health awareness and quality of life, ginseng is widely used in medicine and also has been applied in cosmetics, food, beverage, healthcare, and other industries. The global demand for ginseng exceeds 1,000 tons per year, making outstanding contributions to human health and cultural communication [6–8].

Ginseng is susceptible to diseases and attacks by insects and grass due to its relatively long growth cycle [9–11]. According to statistics, there are more than 40 common diseases and insect pests that can affect the growth of ginseng, and the main diseases include epidemic disease, cataplexy disease, and black spot disease [12, 13]. There are underground and above-ground pests, and underground pests mainly include pupae, gold needles, maggots, and ground tigers [14, 15]. Grasshoppers are the main ground pests. They are widely distributed, have a variety of food habits, and are harmful. Ginseng has holes and notches and is vulnerable to disease. Therefore, pest infestation greatly reduces the yield and quality of ginseng (American ginseng). Pesticides play an important role in improving the yield of crops by preventing and treating plant diseases, pests, and weeds [16–19]. Fluconazole, metalaxyl, propiconazole, flusilazole, azoxystrobin, and other pesticides are usually used to prevent and treat diseases and pests of ginseng [16, 20]. However, due to the unreasonable application of pesticides, accumulation of pesticide residues in ginseng has caused concern worldwide.

Accurate and efficient detection methods provide the basis for qualitative and quantitative analysis of pesticides in ginseng. Some studies have reported the detection method of one or a class of pesticides in ginseng [21, 22]. For example, Park et al. established the multiresidue analysis method for fungicides in ginseng [23]. To date, there have been many detection methods for pesticides applied on ginseng which have not been established. The detection methods for azoxystrobin and trifloxystrobin mainly focused on vegetables and fruits, and there was no standard method for residue detection of the above-mentioned pesticides in ginseng [24].

In this study, a solid-phase extraction technique was developed to simultaneously detect 31 pesticides in ginseng by liquid chromatography-tandem mass spectrometry (LC-MS), including 11 triazole fungicides, 7 methoxyacrylate fungicides, 1 urethane fungicide, 6 amide fungicides, 1 aniline fungicide, 3 anilinopyrimidine fungicides, 1 neonicotinoid insecticide, and toxic metabolites of fluconazole. This research can provide technical support for the detection of multiple residues of pesticides in ginseng. Moreover, this method can improve analysis efficiency and accuracy and shorten the analysis time.

## 2. Materials and Methods

### 2.1. Chemicals and Reagents

LC-grade acetonitrile and methanol were purchased from TEDIA (Fairfield, USA). Cleanert NH_2_-SPE (500 mg/6 ml), Cleanert PSA (500 mg/6 ml), Cleanert PestiCarb SPE (500 mg/6 ml), and Cleanert PestiCarb-NH_2_ SPE (500 mg/6 ml) were provided by Agilent. Standards including Thiamethoxam (purity 99.0%), Triflumizole Metabolite (purity 99.5%), Cymoxanil (purity 99.5%), Flumorph (purity 98.0%), Metalaxyl (purity 99.0%), Dimethomorph (purity 99.0%), Pyrimethanil (purity 98.0%), Epoxiconazole (purity 98.8%), Flusilazole (purity 98.0%), Mepanipyrim (purity 99.5%), Diniconazole (purity 99.0%), Propiconazole (purity 99.0%), Dimoxystrobin (purity 99.0%), Fluoxastrobin (purity 99.3%), Picoxystrobin (purity 99.0%), and Pyraclostrobin (purity 99.0%) were obtained from Dr. Ehrenstorfer GmbH. Myclobutanil (purity 98.6%), Triadimefon (purity 99.5%), Tebuconazole (purity 98.8%), Mandipropamid (purity 99.5%), Hexaconazole (purity 99.5%), Cyprodinil (purity 99.5%), Difenoconazole (purity 99.5%), Kresoxim-methyl (purity 99.5%), Procymidone (purity 99.5%), and Fluazinam (purity 99.5%) were provided by CHEM CHESTER. Paclobutrazol (purity 99.4%), Diethofencarb (purity 99.5%), Azoxystrobin (purity 99.5%), Trifloxystrobin (purity 98.8%), and Triflumizole (purity 99.5%) were purchased from DIKMA. Sodium chloride, anhydrous magnesium sulfate, ammonium acetate, and other analytical-grade reagents were acquired through commercial sources.

### 2.2. Preparation of Stock Standards

Stock standards (1000 mg/L) of individual pesticides were prepared using methanol. An appropriate amount of each stock solution was then accurately measured and diluted to mixed standard stock solutions of 100 and 10 mg/L with methanol. A series of working standards of concentrations of 0.005,0.01, 0.02, 0.05, 0.1,0.2, 0.5, and 1.0 mg/L were prepared by diluting with acetonitrile. The stock standards and working solutions were placed in the dark at –18°C and 4°C, respectively.

### 2.3. Extraction of 31 Pesticides from Ginseng Samples

#### 2.3.1. Extraction from Fresh Ginseng

Approximately 20 g of fresh ginseng samples was weighed into a 100 mL beaker followed by addition of 80 mL acetonitrile and 10 mL distilled water and was vortexed for 2 min. The liquid phase was transferred into a 100 mL cylinder containing 7 g NaCl, shaken for 10 min, and allowed to stand for 60 min. Subsequently, 10 mL of acetonitrile was dehydrated into a flask with 5 g of anhydrous Na_2_SO_4_, followed by evaporating to dryness at 40°C.

#### 2.3.2. Extraction from Dry Ginseng

Approximately 2.0 g of powdered ginseng samples was weighed into a 50 mL Teflon centrifugal tube, 20 mL acetonitrile and 5 mL distilled water were added, and the solution was vortexed for 2 min. 2.0 g NaCl and 4.0 g anhydrous sodium sulfate were added to each centrifuge tube and vigorously vortexed for 1 min, followed by centrifugation for 5 min at 5000 rpm. 10 mL of acetonitrile was dehydrated into a flask with 5 g of anhydrous Na_2_SO_4_, followed by evaporating to dryness at 40°C. The residue was dissolved in 2 mL acetonitrile for cleanup.

### 2.4. Cleanup of the Extracted Samples

The residue was dissolved in 2 mL acetonitrile and transferred into a PSA Solid-Phase Extraction (SPE) pretreated with 4 mL acetonitrile/methyl benzene (3 : 1). The column was rinsed with 5 mL acetonitrile/methylbenzene (3 : 1, v/v). The eluant was collected and evaporated at 40°C. The extract was dissolved with 2 mL acetonitrile and filtered through a 0.22 *μ*m nylon syringe filter for high-performance liquid chromatography with tandem mass spectrometry (HPLC-MS/MS).

### 2.5. HPLC-MS/MS Analysis

The 31 pesticides were separated and quantified by liquid chromatography using the Agilent 1260 high performance liquid chromatograph (Agilent, California, USA) in tandem with Agilent 6460 MS/MS systems (Agilent, California, USA) and Agilent Zorbax RRHD Eclipse Plus C18 column (3.0 × 100 mm 1.8 *µ*m) (Agilent, California, USA). The optimal baseline separation was obtained with 0.1% formic acid in a 5 mM ammonium acetate aqueous solution (A) and acetonitrile (B) with a flow rate of 0.4 mL/min and an injection volume of 5 *μ*L at a column temperature of 30°C. The gradient program is shown in Table S1.

The analysis was performed in both positive and negative ionization modes. The parameters were as follows: source voltages capillary, 3.50 kV; atomization and drying gas, 99.95% nitrogen; collision gas, 99.99% nitrogen; and desolventizer tube temperature, 400°C. All parameters for the multiple reaction mode (MRM) transitions, cone voltage, and collision energy were optimized to obtain the highest sensitivity and resolution.

### 2.6. Method Validation

The linearity, matrix effect, precision, accuracy, limit of detection (LOD), and limit of quantitation (LOQ) were used to evaluate the feasibility of the method for simultaneous determination of 31 pesticides in ginseng. Linearity was assessed by the linear regression of peak areas versus the concentration. The matrix effect should be ignored in the slope ratios of matrix/solvent, ranging from 0.90 to 1.10 [25]. The LOD and LOQ were defined as the concentration that produced a signal-to-noise ratio (S/N) of 3 and 10, respectively.

Accuracy and precision were evaluated by recoveries and interday and intraday relative standard deviations (RSDs) of five spiked samples replicated at three concentrations over three continuous days. The standard solutions were prepared for each pesticide at 0.01, 0.1, and 1.0 mg/kg; these were used to spike the blank samples of ginseng. The spiked samples were shaken for 1 min and then left to sit for 2 h. The extraction and cleanup were performed as described above.

### 2.7. Analysis of Real Samples

The established method was applied to analyze the real samples. The ginseng samples including fresh ginseng and dried ginseng were sampled from market sampling and ginseng planting base in Jilin Province (Ji'an, Baishan, Fusong, and Huanren). All the real samples were extracted and cleanup was performed as described above.

## 3. Results and Discussion

### 3.1. Optimization of Separation and Detection Conditions

**Figure 1:**
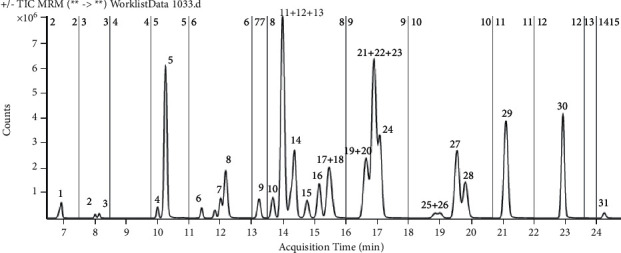
High-performance liquid chromatography multiple reaction mode (HPLC-MRM) chromatogram of 1 mg/L of 31 pesticide standards.

In this study, a series of columns including C8, C18, XDB-CN, and NH_2_ were used to separate the 31 pesticides. Optimal separation was obtained by using Agilent Zorbax RRHD Eclipse Plus C18 (3.0 × 100 mm 1.8 µm) column with 0.1% formic acid in a 5 mM ammonium acetate aqueous solution and acetonitrile as the mobile phase. As shown in Figure 1, all the 31 pesticides were detected within 25 min. Only qualitative and quantitative analysis of Fluazinam was performed in negative ion mode, and the remaining 30 pesticides were detected in the positive ion mode. Precursor ions were selected by the MS Scan mode and the daughter ions were confirmed by the Secondary scan mode. Pesticides were grouped in the order of retention time, the multi-reaction monitoring scanning methods were edited, and segmented scanning to increase the number of ion scans per unit time and improve detection sensitivity was performed. The optical precursor ions, daughter ions, fragmentor, and collision energy are shown in Table 1.

### 3.2. Selection of the Extraction Solvent

MeCN, acetone, and EtA care are usually used as the extraction solvents in the multiresidue analysis of a wide range of pesticides with high recoveries [17–20]. Compared with EtAc and acetone, MeCN is less effective in waxes, fat, and lipophilic pigments [16]. In addition, MeCN is not compatible with nonpolar solvents (hexane), which can effectively remove lipophilic components [21]. MeCN is compatible with GC applications, and, because of its low viscosity and intermediate polarity, it is very useful in reversed-phase liquid chromatography (LC) and SPE applications. Therefore, MeCN was selected as the extraction solvent in this study. According to previous reports, acidified acetonitrile can improve the recovery of acidic pesticides [22–24]; thus, both acetic acid and hydrochloric acid were used to adjust the pH of MeCN from 6 to 2. However, the recovery results indicated that acidified acetonitrile can improve the recovery of acidic pesticides, and the recovery of basic pesticides (such as imidamide and pyrimidine) is less than 60%.

### 3.3. Selection of Solid-Phase Extraction Columns

Ginseng has a complex composition and contains a large number of impurities, such as organic acids, volatile oils, and saponins. In order to obtain a good purification, a solid-phase extraction column with better ability to remove polar impurities was selected for purification. Different solid-phase extraction columns including PSA, NH_2_, Carb, and Carb-NH_2_ column were used to purify the ginseng samples with their respective methods. The results showed that all the good purification results could be obtained with the PSA, Carb-NH_2_, and Carb column. After purification with NH_2_ column, the final solution was cloudy. However, the recoveries ranged from 50% to 70% after purification with the Carb-NH_2_ and Carb column. For the PSA column, the recoveries of all 31 pesticides were greater than 75%. Thus, the PSA column was the most suitable choice.

### 3.4. Method Validation

All the 31 pesticides showed a good linearity in solvent, ranging from 0.005 to 1.0 mg/kg with an *R*^2^ > 0.9921. In this study, when the matrix effect of the pesticide is between 0.9 and 1.1, the samples are calibrated with the standard curve, and others are calibrated with the matrix standard curve. The LOD and LOQ for 31 pesticides in ginseng were 4.4×10^−5^–1.6×10^−2 ^mg/kg and 9.0×10^−5^–3.2×10^−1 ^mg/kg, respectively (Table 2). Excellent accuracy and precision of 31 pesticides were obtained in ginseng. Mean recoveries ranged from 72.1% to 111.6% with 0.9–14.9% of intraday (*n* = 5) RSD and 0.6–7.1% of interday (*n* = 5) RSD. These results indicated that this method could be applied to the simultaneous determination of the 31 pesticides in ginseng (Table 3).

### 3.5. Analysis of Real Samples

The developed simultaneous determination method was applied for determination of 31 pesticides in real samples. In order to ensure the authenticity of the test results during the analysis process, the specificity of the analytical method was confirmed by injecting the quality control samples with each batch of the real samples. All the determination results are shown in Tables S2to S6. Apart from procymidone, no other pesticides were detected in dried ginseng and fresh ginseng. However, the residual concentrations of procymidone in dried and fresh ginseng were below the MRL value from EU. Procymidone, Tebuconazole, Azoxystrobin, Mandipropamid, and Cyprodinil were detected on the fresh ginseng from the planting base. Twelve pesticides, Procymidone, Propiconazole, Tebuconazole, Difenoconazole, Azoxystrobin, Pyraclostrobin, Diethofencarb, Mandipropamid, Cyprodinil, Pyrimethanil, Thiamethoxam, and Dimethomorph, were detected on the dried ginseng from the planting base. The residual concentrations of these pesticides were below the MRL from EU except Procymidone, Tebuconazole, Mandipropamid, and Cyprodinil. These measurement data show that there is pesticide residue pollution in the ginseng in China's current market, which has also led to the frequent return of China's exported ginseng. Therefore, the relevant testing departments should increase the testing of ginseng, strictly check the unqualified ginseng, prohibit its circulation in the market, and ensure China's international reputation to improve import and export trade.

## 4. Conclusions

In this study, a novel analysis method was established for the simultaneous determination of 31 pesticides in ginseng by HPLC-MS/MS. Compared with other methods, this method can simultaneously detect 31 pesticides in fresh ginseng and dried ginseng, with good resolution and sensitivity. The advantages of this method include simple pretreatment, short sample processing time, fast detection speed, and effective elimination of complex matrix interferences of ginseng samples. At the 0.01, 0.1, and 1.0 mg/kg spiked concentrations, the main average recoveries ranged from 72.1 to 111.6%, with RSD values of 1.3–12.2% in fresh ginseng and from 74.3 to 108.3%, with RSD values of 0.9–14.9% in dry ginseng. The recovery and accuracy of the method meet the requirements of pesticide residue analysis, and it is suitable for the simultaneous detection of multiple pesticides in ginseng. The data from the analysis of real samples indicated that there is pesticide residue pollution in the ginseng in China's current market. Although reasonable application of pesticides is essential, this situation must be carefully monitored.

## Figures and Tables

**Table 1:** Mass spectrometry conditions for 31 pesticides.

Compounds	Precursor ion (m/z)	Product ion (m/z)	Fragmentor (V)	Collision energy (V)
Thiamethoxam	292	181	80	20
		211	80	10
Triflumizole metabolite	295	277.9	110	3
		196	110	3
Cymoxanil	199.1	128.1	80	15
		111.1	80	20
Flumorph	371.9	284.9	50	14
		164.8	50	26
Metalaxyl	280	192	120	15
		220	120	20
Dimethomorph	388	301	120	20
		165	120	25
Paclobutrazol	294	70	100	15
		125	100	25
Pyrimethanil	200	107	120	25
		183	120	25
Diethofencarb	268	152	80	20
		226	80	5
Myclobutanil	289	70	120	15
		125	120	20
Azoxystrobin	404	372	120	10
		344	120	15
Epoxiconazole	330	121	120	20
		141	120	20
Triadimefon	294	69	100	20
		197	100	15
Mandipropamid	412.1	328.2	110	8
		125	110	35
Tebuconazole	308	70	100	25
		125	100	25
Flusilazole	316	165	120	20
		247	120	15
Mepanipyrim	224	106	120	25
		77	120	30
Hexaconazole	314	70	120	20
		159	120	20
Diniconazole	326	70	120	25
		159	120	30
Cyprodinil	226	93	120	40
		108	120	30
Procymidone	284.1	256.1	140	10
		145	140	45
Propiconazole	342.1	205	130	10
		159	130	30
Dimoxystrobin	326.9	115.9	99	22
		205	99	5
Fluoxastrobin	459	120	110	9
		137	110	9
Difenoconazole	406	251	160	20
		337	160	15
Kresoxim-methyl	314	131	77	5
		116.1	77	21
Picoxystrobin	368.17	205.13	40	8
		145.12	40	19
Triflumizole	346	73	80	10
		278	80	5
Pyraclostrobin	388	163	20	10
		194	20	10
Trifloxystrobin	409	186	120	15
		206	120	10
Fluazinam	462.9	415.9	120	20
		398	120	15

**Table 2:** Retention time, regression equation, correlation coefficient, linear range, and determination limit of 31 pesticides.

Compounds	Retention time (min)	Regression equation (X, mg/L)	Correlation coefficient (r)	Limit of detection (mg)	Limit of quantitation (mg)	Me
Thiamethoxam	6.947	*y* = 3E + 06x + 113710	0.9944	4.0 × 10^−4^	8.3 × 10^−4^	0.90
Triflumizole metabolite	8.016	*y* = 1E + 06x + 9183.9	0.9962	9.0 × 10^−2^	7.0 × 10^−3^	0.97
Cymoxanil	8.144	*y* = 2E + 06x + 12402	0.9966	6.5 × 10^−4^	3.2 × 10^−4^	0.93
Flumorph	10.008	*y* = 2E + 06x + 1207.1	0.9938	4.2 × 10^−4^	1.0 × 10^−4^	1.00
Metalaxyl	10.263	*y* = 2E + 07x + 3019935	0.9994	2.4 × 10^−4^	8.0 × 10^−4^	1.00
Dimethomorph	11.407	*y* = 8E + 06x－21217	0.9921	3.6 × 10^−3^	8.7 × 10^−4^	1.00
Paclobutrazol	12.022	*y* = 7E + 06x + 93163	0.9964	1.4 × 10^−3^	2.4 × 10^−3^	1.00
Pyrimethanil	12.181	*y* = 1E + 07x + 114381	0.9958	2.0 × 10^−4^	2.6 × 10^−3^	1.00
Diethofencarb	13.246	*y* = 2E + 07x + 428718	0.9944	1.0 × 10^−3^	8.7 × 10^−5^	1.00
Myclobutanil	13.694	*y* = 1E + 07x - 22592	0.9947	1.2 × 10^−3^	3.7 × 10^−3^	1.00
Azoxystrobin	13.994	*y* = 7E + 07x + 3322736.6	0.9990	7.9 × 10^−4^	3.3 × 10^−4^	1.00
Epoxiconazole	14.050	*y* = 1E + 07x + 519680	0.9971	8.0 × 10^−4^	9.0 × 10^−4^	1.00
Triadimefon	14.264	*y* = 7E + 06x + 45104	0.9990	1.6 × 10^−4^	8.0 × 10^−5^	0.90
Mandipropamid	14.377	*y* = 2E + 07x + 786506	0.9952	1.8 × 10^−4^	1.9 × 10^−5^	1.00
Tebuconazole	14.779	*y* = 1E + 07x－65825	0.9948	1.2 × 10^−3^	7.3 × 10^−3^	1.00
Flusilazole	15.161	*y* = 5E + 06x + 31258	0.9951	1.6 × 10^−4^	1.7 × 10^−3^	1.00
Mepanipyrim	15.475	*y* = 6E + 06x + 406501	0.9972	4.4 × 10^−5^	3.2 × 10^−3^	1.00
Hexaconazole	15.558	*y* = 3E + 06x + 17667	0.9957	1.1 × 10^−3^	1.3 × 10^−2^	1.00
Diniconazole	16.643	*y* = 1E + 07x－65825	0.9966	5.2 × 10^−4^	1.5 × 10^−2^	1.00
Cyprodinil	16.650	*y* = 2E + 07x + 716715	0.9959	4.9 × 10^−3^	2.5 × 10^−4^	1.00
Procymidone	16.673	*y* = 9E + 06x－3928.2	0.9997	5.4 × 10^−2^	3.2 × 10^−1^	0.96
Propiconazole	16.775	*y* = 1E + 05x + 1071.9	0.9965	1.6 × 10^−2^	4.7 × 10^−3^	0.94
Dimoxystrobin	16.906	*y* = 6E + 06x + 406501	0.9973	3.9 × 10^−4^	2.7 × 10^−3^	1.00
Fluoxastrobin	17.085	*y* = 3E + 07x + 742899	0.9956	2.3 × 10^−4^	1.3 × 10^−4^	0.95
Difenoconazole	18.851	*y* = 2E + 06x－10631	0.9937	7.5 × 10^−3^	2.7 × 10^−3^	0.92
Kresoxim-methyl	19.037	*y* = 2E + 06x + 109945	0.9968	2.3 × 10^−3^	3.3 × 10^−4^	1.00
Picoxystrobin	19.556	*y* = 1E + 07x + 879577	0.9960	7.6 × 10^−5^	9.0 × 10^−5^	1.00
Triflumizole	19.821	*y* = 1E + 07x + 760728	0.9961	1.9 × 10^−4^	4.7 × 10^−4^	0.94
Pyraclostrobin	21.107	*y* = 2E + 07x + 23067.2	0.9981	1.2 × 10^−4^	5.7 × 10^−4^	1.00
Trifloxystrobin	22.925	*y* = 3E + 07x－169245	0.9979	5.1 × 10^−5^	4.7×10–4	1.00
Fluazinam	24.243	*y* = 7E + 06x + 12129	0.9984	2.3 × 10^−4^	5.7 × 10^−4^	1.03

**Table 3:** Recoveries and precision of detection of 31 pesticides in different ginseng samples (*n* = 5).

Compounds	Spiked level (mg/kg)	Average recovery (%) (fresh)	RSD (%)	Average recovery (%) (dry)	RSD (%)
Thiamethoxam	0.01	107.3	1.3	99.7	7.7
	0.1	86.8	2.2	82.3	5.7
	1	91.2	2.3	82.9	10.1
Triflumizole metabolite	0.01	93.2	4.3	76.0	2.2
	0.1	76.7	3.5	82.8	2.7
	1	88.3	4.6	93.0	9.3
Cymoxanil	0.01	97.5	4.6	96.8	7.1
	0.1	104.2	3.8	88.0	4.7
	1	89.1	1.5	90.6	8.1
Flumorph	0.01	89.6	4.6	91.5	10.3
	0.1	100.1	2.3	84.4	2.6
	1	89.1	1.5	90.6	8.1
Metalaxyl	0.01	111.6	3.2	104.4	4.1
	0.1	93.2	8.7	95.7	3.5
	1	93.6	2.3	90.6	10.9
Dimethomorph	0.01	79.9	4.9	91.9	8.0
	0.1	94.4	1.9	78.8	3.0
	1	84.4	2.0	86.9	11.2
Paclobutrazol	0.01	100.3	3.9	85.3	9.9
	0.1	80.3	4.0	86.3	1.9
	1	91.7	2.2	82.6	11.5
Pyrimethanil	0.01	91.7	3.6	103.3	3.2
	0.1	101.9	1.8	98.4	2.5
	1	85.2	3.0	99.6	6.7
Diethofencarb	0.01	88.3	3.0	92.0	10.3
	0.1	101.9	2.7	84.4	3.8
	1	85.2	2.7	88.7	11.4
Myclobutanil	0.01	92.6	2.2	107.9	1.9
	0.1	91.6	2.3	98.6	9.9
	1	85.4	2.4	86.3	11.9
Azoxystrobin	0.01	82.3	5.0	93.3	9.5
	0.1	94.6	1.5	83.4	3.2
	1	87.0	2.6	89.7	13.2
Epoxiconazole	0.01	88.8	4.6	92.0	11.3
	0.1	103.3	1.5	86.5	2.2
	1	87.2	2.3	90.9	12.5
Triadimefon	0.01	86.3	6.3	95.1	8.0
	0.1	99.5	1.6	86.4	2.8
	1	87.2	2.3	90.9	12.5
Mandipropamid	0.01	89.1	4.9	108.0	2.1
	0.1	100.8	2.2	86.3	3.4
	1	86.2	2.6	86.9	14.9
Tebuconazole	0.01	83.0	3.1	92.4	10.5
	0.1	96.8	2.2	82.7	3.1
	1	85.7	1.9	86.3	11.9
Flusilazole	0.01	91.9	3.8	88.9	12.1
	0.1	90.9	3.7	85.6	3.2
	1	90.6	1.9	88.4	10.6
Mepanipyrim	0.01	84.2	4.2	86.4	10.5
	0.1	96.7	2.0	83.4	2.7
	1	84.9	2.1	87.5	11.3
Hexaconazole	0.01	100.9	8.9	91.2	7.0
	0.1	95.9	2.1	81.3	4.4
	1	89.6	1.8	84.1	11.6
Diniconazole	0.01	81.5	4.4	89.1	11.5
	0.1	98.6	2.0	84.1	3.3
	1	86.2	1.6	87.0	13.6
Cyprodinil	0.01	101.5	12.2	94.3	10.8
	0.1	92.7	8.7	95.8	2.7
	1	88.9	1.9	90.5	11.7
Procymidone	0.01	101.7	3.8	105.7	2.0
	0.1	105.4	2.1	92.2	4.9
	1	87.3	2.8	87.3	10.3
Propiconazole	0.01	84.3	4.5	90.5	10.9
	0.1	98.5	2.1	85.8	4.4
	1	88.9	1.9	90.3	10.8
Dimoxystrobin	0.01	86.6	4.6	91.9	9.2
	0.1	101.1	1.4	87.6	3.8
	1	88.9	1.9	90.3	10.8
Fluoxastrobin	0.01	92.5	3.6	108.3	0.9
	0.1	90.8	3.1	89.1	3.0
	1	83.0	2.1	88.2	13.8
Difenoconazole	0.01	88.8	3.2	90.3	8.5
	0.1	90.5	3.0	87.3	3.9
	1	88.1	1.9	87.3	10.6
Kresoxim-methyl	0.01	88.9	2.8	88.5	13.7
	0.1	93.1	1.9	84.8	2.3
	1	88.5	2.0	88.8	11.6
Picoxystrobin	0.01	78.7	3.1	85.3	11.8
	0.1	72.1	1.6	74.3	3.4
	1	82.4	2.2	78.4	12.1
Triflumizole	0.01	94.1	4.5	96.8	5.7
	0.1	100.0	2.7	85.7	3.1
	1	93.5	1.9	95.6	7.7
Pyraclostrobin	0.01	86.2	4.3	85.1	12.0
	0.1	96.4	2.6	92.6	2.7
	1	104.2	3.0	97.8	4.9
Trifloxystrobin	0.01	87.2	3.4	91.4	10.5
	0.1	87.2	3.7	83.9	2.8
	1	104.2	3.0	97.8	4.9
Fluazinam	0.01	93.0	4.4	98.5	2.7
	0.1	91.3	2.9	81.1	2.5
	1	89.7	2.8	94.9	2.1

## Data Availability

Processed data used to support the findings of this study are included within the article. Some of the data related to the validation of the developed method are also included within the article, while others are included as Supplementary Materials (S1–S6).

## Supplementary Materials

Supplementary Materials Table S1: the gradient program for simultaneous determination of 31 pesticides. Table S2: pesticide residues in dried ginseng and fresh ginseng from market (mg/kg). Table S3: pesticide residues in fresh ginseng from planting base (mg/kg). Table S4: pesticide residues in fresh ginseng from planting base (mg/kg). Table S5 and Table S6: pesticide residues in dried ginseng from planting base (mg/kg).
