# Supplementary material for: A Novel Analysis Method for Simultaneous Determination of 31 Pesticides by High-Performance Liquid Chromatography-Tandem Mass Spectrometry in Ginseng
Source: J Anal Methods Chem. 2022 Feb 16;2022:4208243. doi: 10.1155/2022/4208243 (PMC8866027; doi:10.1155/2022/4208243)
Supplement: Supplementary Materials — Table S1: the gradient program for simultaneous determination of 31 pesticides. Table S2: pesticide residues in dried ginseng and fresh ginseng from market (mg/kg). Table S3: pesticide residues in fresh ginseng from planting base (mg/kg). Table S4: pesticide residues in fresh ginseng from planting base (mg/kg). Table S5 and Table S6: pesticide residues in dried ginseng from planting base (mg/kg). [file 4208243.f1.doc]

**Supporting Information**

**A novel analysis method for simultaneous determination 31 pesticides by high-performance liquid chromatography−tandem mass spectrometry in Ginseng**

Table S1 The gradient program for simultaneous determination 31 pesticides

| Time（min） | Water(%) | Acetonitrile(%) |
| --- | --- | --- |
| 0 | 99 | 1 |
| 4 | 50 | 50 |
| 15 | 40 | 60 |
| 20 | 20 | 80 |
| 25 | 1 | 99 |
| 30 | 1 | 99 |
| 30.1 | 99 | 1 |

Table S2 Pesticide residues in dried ginseng and fresh ginseng from mark (mg/kg)

| Pesticides | Dried ginseng | | | Fresh ginseng | | |
| --- | --- | --- | --- | --- | --- | --- |
|  | Sample 1 | Sample 2 | Sample 3 | Sample 1 | Sample 2 | Sample 3 |
| Triflumizole | ≤0.01 | ≤0.01 | ≤0.01 | ≤0.01 | ≤0.01 | ≤0.01 |
| Propiconazole | ≤0.01 | ≤0.01 | ≤0.01 | ≤0.01 | ≤0.01 | ≤0.01 |
| Flusilazole | ≤0.01 | ≤0.01 | ≤0.01 | ≤0.01 | ≤0.01 | ≤0.01 |
| Myclobutanil | ≤0.01 | ≤0.01 | ≤0.01 | ≤0.01 | ≤0.01 | ≤0.01 |
| Tebuconazole | ≤0.01 | ≤0.01 | ≤0.01 | ≤0.01 | ≤0.01 | ≤0.01 |
| Hexaconazole | ≤0.01 | ≤0.01 | ≤0.01 | ≤0.01 | ≤0.01 | ≤0.01 |
| Triadimefon | ≤0.01 | ≤0.01 | ≤0.01 | ≤0.01 | ≤0.01 | ≤0.01 |
| Epoxiconazole | ≤0.01 | ≤0.01 | ≤0.01 | ≤0.01 | ≤0.01 | ≤0.01 |
| Diniconazole | ≤0.01 | ≤0.01 | ≤0.01 | ≤0.01 | ≤0.01 | ≤0.01 |
| Difenoconazole | ≤0.01 | ≤0.01 | ≤0.01 | ≤0.01 | ≤0.01 | ≤0.01 |
| Azoxystrobin | ≤0.01 | ≤0.01 | ≤0.01 | ≤0.01 | ≤0.01 | ≤0.01 |
| Kresoxim-methyl | ≤0.01 | ≤0.01 | ≤0.01 | ≤0.01 | ≤0.01 | ≤0.01 |
| Pyraclostrobin | ≤0.01 | ≤0.01 | ≤0.01 | ≤0.01 | ≤0.01 | ≤0.01 |
| Trifloxystrobin | ≤0.01 | ≤0.01 | ≤0.01 | ≤0.01 | ≤0.01 | ≤0.01 |
| Picoxystrobin | ≤0.01 | ≤0.01 | ≤0.01 | ≤0.01 | ≤0.01 | ≤0.01 |
| Fluoxastrobin | ≤0.01 | ≤0.01 | ≤0.01 | ≤0.01 | ≤0.01 | ≤0.01 |
| Mepanipyrim | ≤0.01 | ≤0.01 | ≤0.01 | ≤0.01 | ≤0.01 | ≤0.01 |
| Diethofencarb | ≤0.01 | ≤0.01 | ≤0.01 | ≤0.01 | ≤0.01 | ≤0.01 |
| Thiamethoxam | ≤0.01 | ≤0.01 | ≤0.01 | ≤0.01 | ≤0.01 | ≤0.01 |
| Metalaxyl | ≤0.01 | ≤0.01 | ≤0.01 | ≤0.01 | ≤0.01 | ≤0.01 |
| Mandipropamid | ≤0.01 | ≤0.01 | ≤0.01 | ≤0.01 | ≤0.01 | ≤0.01 |
| Flumorph | ≤0.01 | ≤0.01 | ≤0.01 | ≤0.01 | ≤0.01 | ≤0.01 |
| Dimethomorph | ≤0.01 | ≤0.01 | ≤0.01 | ≤0.01 | ≤0.01 | ≤0.01 |
| Cyprodinil | ≤0.01 | ≤0.01 | ≤0.01 | ≤0.01 | ≤0.01 | ≤0.01 |
| Pyrimethanil | ≤0.01 | ≤0.01 | ≤0.01 | ≤0.01 | ≤0.01 | ≤0.01 |
| Dimoxystrobin | ≤0.01 | ≤0.01 | ≤0.01 | ≤0.01 | ≤0.01 | ≤0.01 |
| Cymoxanil | ≤0.01 | ≤0.01 | ≤0.01 | ≤0.01 | ≤0.01 | ≤0.01 |
| Procymidone | ≤0.01 | 0.019 | 0.065 | 0.056 | 0.084 | 0.056 |
| Paclobutrazol | ≤0.01 | ≤0.01 | ≤0.01 | ≤0.01 | ≤0.01 | ≤0.01 |
| Fluazinam | ≤0.01 | ≤0.01 | ≤0.01 | ≤0.01 | ≤0.01 | ≤0.01 |
| Triflumizole Metabolite Standard | ≤0.01 | ≤0.01 | ≤0.01 | ≤0.01 | ≤0.01 | ≤0.01 |

Table S3 Pesticide residues in fresh ginseng from planting base (mg/kg)

| Pesticides | FuSong | | | Jian | | |
| --- | --- | --- | --- | --- | --- | --- |
|  | Sample 1 | Sample 2 | Sample 3 | Sample 1 | Sample 2 | Sample 3 |
| Triflumizole | ≤0.01 | ≤0.01 | ≤0.01 | ≤0.01 | ≤0.01 | ≤0.01 |
| Propiconazole | ≤0.01 | ≤0.01 | ≤0.01 | ≤0.01 | ≤0.01 | ≤0.01 |
| Flusilazole | ≤0.01 | ≤0.01 | ≤0.01 | ≤0.01 | ≤0.01 | ≤0.01 |
| Myclobutanil | ≤0.01 | ≤0.01 | ≤0.01 | ≤0.01 | ≤0.01 | ≤0.01 |
| Tebuconazole | ≤0.01 | ≤0.01 | ≤0.01 | ≤0.01 | ≤0.01 | 0.019 |
| Hexaconazole | ≤0.01 | ≤0.01 | ≤0.01 | ≤0.01 | ≤0.01 | ≤0.01 |
| Triadimefon | ≤0.01 | ≤0.01 | ≤0.01 | ≤0.01 | ≤0.01 | ≤0.01 |
| Epoxiconazole | ≤0.01 | ≤0.01 | ≤0.01 | ≤0.01 | ≤0.01 | ≤0.01 |
| Diniconazole | ≤0.01 | ≤0.01 | ≤0.01 | ≤0.01 | ≤0.01 | ≤0.01 |
| Difenoconazole | ≤0.01 | ≤0.01 | ≤0.01 | ≤0.01 | ≤0.01 | ≤0.01 |
| Azoxystrobin | 0.013 | ≤0.01 | 0.031 | ≤0.01 | ≤0.01 | ≤0.01 |
| Kresoxim-methyl | ≤0.01 | ≤0.01 | ≤0.01 | ≤0.01 | ≤0.01 | ≤0.01 |
| Pyraclostrobin | ≤0.01 | ≤0.01 | ≤0.01 | ≤0.01 | ≤0.01 | ≤0.01 |
| Trifloxystrobin | ≤0.01 | ≤0.01 | ≤0.01 | ≤0.01 | ≤0.01 | ≤0.01 |
| Picoxystrobin | ≤0.01 | ≤0.01 | ≤0.01 | ≤0.01 | ≤0.01 | ≤0.01 |
| Fluoxastrobin | ≤0.01 | ≤0.01 | ≤0.01 | ≤0.01 | ≤0.01 | ≤0.01 |
| Mepanipyrim | ≤0.01 | ≤0.01 | ≤0.01 | ≤0.01 | ≤0.01 | ≤0.01 |
| Diethofencarb | ≤0.01 | ≤0.01 | ≤0.01 | ≤0.01 | ≤0.01 | ≤0.01 |
| Thiamethoxam | ≤0.01 | ≤0.01 | ≤0.01 | ≤0.01 | ≤0.01 | ≤0.01 |
| Metalaxyl | ≤0.01 | ≤0.01 | ≤0.01 | ≤0.01 | ≤0.01 | ≤0.01 |
| Mandipropamid | 0.075 | 0.031 | ≤0.01 | ≤0.01 | 0.025 | ≤0.01 |
| Flumorph | ≤0.01 | ≤0.01 | ≤0.01 | ≤0.01 | ≤0.01 | ≤0.01 |
| Dimethomorph | ≤0.01 | ≤0.01 | ≤0.01 | ≤0.01 | ≤0.01 | ≤0.01 |
| Cyprodinil | 0.144 | ≤0.01 | ≤0.01 | ≤0.01 | ≤0.01 | 0.014 |
| Pyrimethanil | ≤0.01 | ≤0.01 | ≤0.01 | ≤0.01 | ≤0.01 | ≤0.01 |
| Dimoxystrobin | ≤0.01 | ≤0.01 | ≤0.01 | ≤0.01 | ≤0.01 | ≤0.01 |
| Cymoxanil | ≤0.01 | ≤0.01 | ≤0.01 | ≤0.01 | ≤0.01 | ≤0.01 |
| Procymidone | 0.014 | ≤0.01 | ≤0.01 | ≤0.01 | ≤0.01 | ≤0.01 |
| Paclobutrazol | ≤0.01 | ≤0.01 | ≤0.01 | ≤0.01 | ≤0.01 | ≤0.01 |
| Fluazinam | ≤0.01 | ≤0.01 | ≤0.01 | ≤0.01 | ≤0.01 | ≤0.01 |
| Triflumizole Metabolite Standard | ≤0.01 | ≤0.01 | ≤0.01 | ≤0.01 | ≤0.01 | ≤0.01 |

Table S4 Pesticide residues in fresh ginseng from planting base (mg/kg)

| Pesticides | Baishan | | | Huanheng | | |
| --- | --- | --- | --- | --- | --- | --- |
|  | Sample 1 | Sample 2 | Sample 3 | Sample 1 | Sample 2 | Sample 3 |
| Triflumizole | ≤0.01 | ≤0.01 | ≤0.01 | ≤0.01 | ≤0.01 | ≤0.01 |
| Propiconazole | ≤0.01 | ≤0.01 | ≤0.01 | ≤0.01 | ≤0.01 | ≤0.01 |
| Flusilazole | ≤0.01 | ≤0.01 | ≤0.01 | ≤0.01 | ≤0.01 | ≤0.01 |
| Myclobutanil | ≤0.01 | ≤0.01 | ≤0.01 | ≤0.01 | ≤0.01 | ≤0.01 |
| Tebuconazole | ≤0.01 | ≤0.01 | ≤0.01 | ≤0.01 | ≤0.01 | 0.019 |
| Hexaconazole | ≤0.01 | ≤0.01 | ≤0.01 | ≤0.01 | ≤0.01 | ≤0.01 |
| Triadimefon | ≤0.01 | ≤0.01 | ≤0.01 | ≤0.01 | ≤0.01 | ≤0.01 |
| Epoxiconazole | ≤0.01 | ≤0.01 | ≤0.01 | ≤0.01 | ≤0.01 | ≤0.01 |
| Diniconazole | ≤0.01 | ≤0.01 | ≤0.01 | ≤0.01 | ≤0.01 | ≤0.01 |
| Difenoconazole | ≤0.01 | ≤0.01 | ≤0.01 | ≤0.01 | ≤0.01 | ≤0.01 |
| Azoxystrobin | 0.013 | ≤0.01 | 0.031 | ≤0.01 | ≤0.01 | ≤0.01 |
| Kresoxim-methyl | ≤0.01 | ≤0.01 | ≤0.01 | ≤0.01 | ≤0.01 | ≤0.01 |
| Pyraclostrobin | ≤0.01 | ≤0.01 | ≤0.01 | ≤0.01 | ≤0.01 | ≤0.01 |
| Trifloxystrobin | ≤0.01 | ≤0.01 | ≤0.01 | ≤0.01 | ≤0.01 | ≤0.01 |
| Picoxystrobin | ≤0.01 | ≤0.01 | ≤0.01 | ≤0.01 | ≤0.01 | ≤0.01 |
| Fluoxastrobin | ≤0.01 | ≤0.01 | ≤0.01 | ≤0.01 | ≤0.01 | ≤0.01 |
| Mepanipyrim | ≤0.01 | ≤0.01 | ≤0.01 | ≤0.01 | ≤0.01 | ≤0.01 |
| Diethofencarb | ≤0.01 | ≤0.01 | ≤0.01 | ≤0.01 | ≤0.01 | ≤0.01 |
| Thiamethoxam | ≤0.01 | ≤0.01 | ≤0.01 | ≤0.01 | ≤0.01 | ≤0.01 |
| Metalaxyl | ≤0.01 | ≤0.01 | ≤0.01 | ≤0.01 | ≤0.01 | ≤0.01 |
| Mandipropamid | 0.075 | 0.031 | ≤0.01 | ≤0.01 | 0.025 | ≤0.01 |
| Flumorph | ≤0.01 | ≤0.01 | ≤0.01 | ≤0.01 | ≤0.01 | ≤0.01 |
| Dimethomorph | ≤0.01 | ≤0.01 | ≤0.01 | ≤0.01 | ≤0.01 | ≤0.01 |
| Cyprodinil | 0.144 | ≤0.01 | ≤0.01 | ≤0.01 | ≤0.01 | 0.014 |
| Pyrimethanil | ≤0.01 | ≤0.01 | ≤0.01 | ≤0.01 | ≤0.01 | ≤0.01 |
| Dimoxystrobin | ≤0.01 | ≤0.01 | ≤0.01 | ≤0.01 | ≤0.01 | ≤0.01 |
| Cymoxanil | ≤0.01 | ≤0.01 | ≤0.01 | ≤0.01 | ≤0.01 | ≤0.01 |
| Procymidone | ≤0.01 | ≤0.01 | ≤0.01 | 0.221 | 0.068 | ≤0.01 |
| Paclobutrazol | ≤0.01 | ≤0.01 | ≤0.01 | ≤0.01 | ≤0.01 | ≤0.01 |
| Fluazinam | ≤0.01 | ≤0.01 | ≤0.01 | ≤0.01 | ≤0.01 | ≤0.01 |
| Triflumizole Metabolite Standard | ≤0.01 | ≤0.01 | ≤0.01 | ≤0.01 | ≤0.01 | ≤0.01 |

Table S5 Pesticide residues in dried ginseng from planting base (mg/kg)

| Pesticides | Fusong | | | Jian | | |
| --- | --- | --- | --- | --- | --- | --- |
|  | Sample 1 | Sample 2 | Sample 3 | Sample 1 | Sample 2 | Sample 3 |
| Triflumizole | ≤0.01 | ≤0.01 | ≤0.01 | ≤0.01 | ≤0.01 | ≤0.01 |
| Propiconazole | ≤0.01 | ≤0.01 | 0.013 | ≤0.01 | 0.013 | ≤0.01 |
| Flusilazole | ≤0.01 | ≤0.01 | ≤0.01 | ≤0.01 | ≤0.01 | ≤0.01 |
| Myclobutanil | ≤0.01 | ≤0.01 | ≤0.01 | ≤0.01 | ≤0.01 | ≤0.01 |
| Tebuconazole | ≤0.01 | ≤0.01 | 0.019 | ≤0.01 | ≤0.01 | ≤0.01 |
| Hexaconazole | ≤0.01 | ≤0.01 | ≤0.01 | ≤0.01 | ≤0.01 | ≤0.01 |
| Triadimefon | ≤0.01 | ≤0.01 | ≤0.01 | ≤0.01 | ≤0.01 | ≤0.01 |
| Epoxiconazole | ≤0.01 | ≤0.01 | ≤0.01 | ≤0.01 | ≤0.01 | ≤0.01 |
| Diniconazole | ≤0.01 | ≤0.01 | ≤0.01 | ≤0.01 | ≤0.01 | ≤0.01 |
| Difenoconazole | 0.011 | ≤0.01 | 0.012 | ≤0.01 | ≤0.01 | ≤0.01 |
| Azoxystrobin | 0.059 | ≤0.01 | 0.012 | 0.030 | ≤0.01 | ≤0.01 |
| Kresoxim-methyl | ≤0.01 | ≤0.01 | ≤0.01 | ≤0.01 | ≤0.01 | ≤0.01 |
| Pyraclostrobin | ≤0.01 | ≤0.01 | ≤0.01 | ≤0.01 | ≤0.01 | ≤0.01 |
| Trifloxystrobin | ≤0.01 | ≤0.01 | ≤0.01 | ≤0.01 | ≤0.01 | ≤0.01 |
| Picoxystrobin | ≤0.01 | ≤0.01 | ≤0.01 | ≤0.01 | ≤0.01 | ≤0.01 |
| Fluoxastrobin | ≤0.01 | ≤0.01 | ≤0.01 | ≤0.01 | ≤0.01 | ≤0.01 |
| Mepanipyrim | ≤0.01 | ≤0.01 | ≤0.01 | ≤0.01 | ≤0.01 | ≤0.01 |
| Diethofencarb | ≤0.01 | ≤0.01 | ≤0.01 | ≤0.01 | ≤0.01 | ≤0.01 |
| Thiamethoxam | ≤0.01 | 0.017 | ≤0.01 | ≤0.01 | ≤0.01 | ≤0.01 |
| Metalaxyl | ≤0.01 | ≤0.01 | ≤0.01 | ≤0.01 | ≤0.01 | ≤0.01 |
| Mandipropamid | ≤0.01 | ≤0.01 | ≤0.01 | ≤0.01 | ≤0.01 | ≤0.01 |
| Flumorph | ≤0.01 | ≤0.01 | ≤0.01 | ≤0.01 | ≤0.01 | ≤0.01 |
| Dimethomorph | ≤0.01 | ≤0.01 | ≤0.01 | ≤0.01 | ≤0.01 | ≤0.01 |
| Cyprodinil | 0.05 | ≤0.01 | ≤0.01 | 0.062 | ≤0.01 | ≤0.01 |
| Pyrimethanil | ≤0.01 | ≤0.01 | 0.019 | ≤0.01 | ≤0.01 | ≤0.01 |
| Dimoxystrobin | ≤0.01 | ≤0.01 | ≤0.01 | ≤0.01 | ≤0.01 | ≤0.01 |
| Cymoxanil | ≤0.01 | ≤0.01 | ≤0.01 | ≤0.01 | ≤0.01 | ≤0.01 |
| Procymidone | 0.026 | 0.014 | 0.78 | 0.014 | ≤0.01 | 0.191 |
| Paclobutrazol | ≤0.01 | ≤0.01 | ≤0.01 | ≤0.01 | ≤0.01 | ≤0.01 |
| Fluazinam | ≤0.01 | ≤0.01 | ≤0.01 | ≤0.01 | ≤0.01 | ≤0.01 |
| Triflumizole Metabolite Standard | ≤0.01 | ≤0.01 | ≤0.01 | ≤0.01 | ≤0.01 | ≤0.01 |

Table S6 Pesticide residues in dried ginseng from planting base (mg/kg)

| Pesticides | Baishan | | | Huanheng | | |
| --- | --- | --- | --- | --- | --- | --- |
|  | Sample 1 | Sample 2 | Sample 3 | Sample 1 | Sample 2 | Sample 3 |
| Triflumizole | ≤0.01 | ≤0.01 | ≤0.01 | ≤0.01 | ≤0.01 | ≤0.01 |
| Propiconazole | ≤0.01 | ≤0.01 | ≤0.01 | ≤0.01 | ≤0.01 | ≤0.01 |
| Flusilazole | ≤0.01 | ≤0.01 | ≤0.01 | ≤0.01 | ≤0.01 | ≤0.01 |
| Myclobutanil | ≤0.01 | ≤0.01 | ≤0.01 | ≤0.01 | ≤0.01 | ≤0.01 |
| Tebuconazole | ≤0.01 | ≤0.01 | 0.05 | 0.019 | ≤0.01 | 0.170 |
| Hexaconazole | ≤0.01 | ≤0.01 | ≤0.01 | ≤0.01 | ≤0.01 | ≤0.01 |
| Triadimefon | ≤0.01 | ≤0.01 | ≤0.01 | ≤0.01 | ≤0.01 | ≤0.01 |
| Epoxiconazole | ≤0.01 | ≤0.01 | ≤0.01 | ≤0.01 | ≤0.01 | ≤0.01 |
| Diniconazole | ≤0.01 | ≤0.01 | ≤0.01 | ≤0.01 | ≤0.01 | ≤0.01 |
| Difenoconazole | ≤0.01 | ≤0.01 | ≤0.01 | ≤0.01 | ≤0.01 | ≤0.01 |
| Azoxystrobin | ≤0.01 | ≤0.01 | ≤0.01 | 0.162 | ≤0.01 | 0.038 |
| Kresoxim-methyl | ≤0.01 | ≤0.01 | ≤0.01 | ≤0.01 | ≤0.01 | ≤0.01 |
| Pyraclostrobin | ≤0.01 | ≤0.01 | ≤0.01 | ≤0.01 | ≤0.01 | 0.075 |
| Trifloxystrobin | ≤0.01 | ≤0.01 | ≤0.01 | ≤0.01 | ≤0.01 | ≤0.01 |
| Picoxystrobin | ≤0.01 | ≤0.01 | ≤0.01 | ≤0.01 | ≤0.01 | ≤0.01 |
| Fluoxastrobin | ≤0.01 | ≤0.01 | ≤0.01 | ≤0.01 | ≤0.01 | ≤0.01 |
| Mepanipyrim | ≤0.01 | ≤0.01 | ≤0.01 | ≤0.01 | ≤0.01 | ≤0.01 |
| Diethofencarb | 0.022 | ≤0.01 | ≤0.01 | ≤0.01 | ≤0.01 | ≤0.01 |
| Thiamethoxam | ≤0.01 | ≤0.01 | ≤0.01 | 0.010 | ≤0.01 | ≤0.01 |
| Metalaxyl | ≤0.01 | ≤0.01 | ≤0.01 | ≤0.01 | ≤0.01 | ≤0.01 |
| Mandipropamid | 0.110 | 0.90 | ≤0.01 | ≤0.01 | ≤0.01 | ≤0.01 |
| Flumorph | ≤0.01 | ≤0.01 | ≤0.01 | ≤0.01 | ≤0.01 | ≤0.01 |
| Dimethomorph | ≤0.01 | ≤0.01 | ≤0.01 | ≤0.01 | ≤0.01 | 0.013 |
| Cyprodinil | 0.014 | ≤0.01 | 0.051 | ≤0.01 | ≤0.01 | ≤0.01 |
| Pyrimethanil | ≤0.01 | ≤0.01 | ≤0.01 | ≤0.01 | ≤0.01 | ≤0.01 |
| Dimoxystrobin | ≤0.01 | ≤0.01 | ≤0.01 | ≤0.01 | ≤0.01 | ≤0.01 |
| Cymoxanil | ≤0.01 | ≤0.01 | ≤0.01 | ≤0.01 | ≤0.01 | ≤0.01 |
| Procymidone | ≤0.01 | ≤0.01 | ≤0.01 | ≤0.01 | ≤0.01 | ≤0.01 |
| Paclobutrazol | ≤0.01 | ≤0.01 | ≤0.01 | ≤0.01 | ≤0.01 | ≤0.01 |
| Fluazinam | ≤0.01 | ≤0.01 | ≤0.01 | ≤0.01 | ≤0.01 | ≤0.01 |
| Triflumizole Metabolite Standard | ≤0.01 | ≤0.01 | ≤0.01 | ≤0.01 | ≤0.01 | ≤0.01 |
